# Influence of cognitive impairment and dementia on oral health and the utilization of dental services: Findings of the Oral Health, Bite force and Dementia Study (OrBiD)

**DOI:** 10.1186/s12903-021-01753-3

**Published:** 2021-08-14

**Authors:** Julia Jockusch, Werner Hopfenmüller, Ina Nitschke

**Affiliations:** 1 University Research Priority Program (URPP), Dynamics of Healthy Aging, Andreasstrasse 15, 8050 Zurich, Switzerland; 2 Institute of Biometry and Clinical Epidemiology (iBikE), Charité - Universitätsmedizin Berlin, corporate member of Freie Universität Berlin, Humboldt-Universität zu Berlin, and Berlin Institute of Health, Campus Charité Mitte, Charitéplatz 1, 10117 Berlin, Germany; 3 Clinic of General, Special Care and Geriatric Dentistry, Center of Dental Medicine, University of Zurich, Plattenstrasse 11, 8032 Zurich, Switzerland; 4 Department of Prosthodontics and Materials Science, Gerodontology Section, Universitätsklinikum Leipzig AöR, Leipzig, Germany

**Keywords:** Oral health, Dementia, Cognitive impairment, Uptake of dental services, DMFT index, Periodontal disease, Bleeding on probing, Oral hygiene index

## Abstract

**Background:**

The aim of this study was to show the influence of cognitive impairment and dementia on oral health and on the utilization of dental services.

**Methods:**

A cross-sectional analyzation of data of the OrBiD (Oral Health, Bite Force and Dementia) pilot study was conducted. 137 subjects were stratified into five dementia groups on the basis of the Mini Mental State Examination (MMSE) (1—no dementia (MMSE 28–30), 2—mild cognitive impairment (MMSE 25–27), 3—mild dementia (MMSE 18–24), 4—moderate dementia (MMSE 10–17), and 5—severe dementia (MMSE < 10)). Information on the utilization of dental services and oral health parameters (DMFT index, degree of restoration, Periodontal Screening Index, Bleeding on Probing, Oral Hygiene Index, Denture Hygiene Index) were collected.

**Results:**

An increase in dementia resulted in significant reduction in utilization. Moreover, with increasing cognitive impairment/dementia there was a significant difference in the number of teeth that were decayed, but not in the number of filled or missing teeth or the DMF/T index itself. With increasing dementia, the degree of restoration decreased and oral/denture hygiene deteriorated significantly. Nevertheless, periodontal therapy was required for all subjects independent of their degree of dementia while bleeding on probing was increasing with increasing dementia.

**Conclusions:**

An influence of cognitive impairment and dementia on oral health and on the utilization of dental services was shown. However, no conclusions about the influence of the utilization behavior of people with dementia on oral health parameters can be drawn. Further longitudinal studies are needed.

*Trial registration* ClinicalTrials.gov NCT03775772. Registered 14th December 2018, https://clinicaltrials.gov/ct2/show/NCT03775772.

## Background

### Demographic shift and consequences for dentistry

Due to demographic change, dentists will also have to focus on the dental care and treatment of the heterogeneous patient group of the old and very old in the future. Dentists can only successfully meet the challenges of this specific patient group with the help of a combination of dental as well as interdisciplinary knowledge (e.g. geriatrics, ethics, nursing sciences, etc.). Furthermore, psycho-social skills such as patient management, communication concepts for dementia, etc. are also required for senior-friendly treatment [1, 2]. Dementia is one of the greatest societal challenges in view of the increasing number of people with the disease. By 2050, around 315’400 people with dementia are expected, with an upward trend [3].

Prevention successes in dentistry result in a higher number of own teeth in the old and very old people. The increasing age of the population along with the occurrence of co-morbidities such as dementia urges us to work for good oral health to prevent pain and emergency treatments in people old and very old people with and without dementia.

### Dementia, its signs and symptoms

Dementia causes the loss of cognitive functions such as thinking, remembering and reasoning. Additionally, behavioral skills also decline. These reduced functions affect skills and abilities (e.g. memory, language skills, visual perception, problem solving, self-management and the ability to concentrate and pay attention) which are essential for the daily life and activities of people [4].

Furthermore, the loss of the control of emotions occurs as well as an alteration in personality. Depending on the severity of dementia, a person can only be slightly affected in its ability to function (mildest stage) or can completely be dependent on others for basic life activities [4]. The Mini-Mental State Examination (MMSE) is often used as a simple geriatric assessment tool to determine cognitive impairment [5].

### Dementia, and its relation to oral health

Knowledge of this patient group in terms of dental care levels, possible prevalence and incidence of oral diseases and treatment options is currently very limited. Due to the limited study data available, there is an urgent need for research in this area. One of the goals should be to develop an approach to dental treatment for this patient group that is both professional-oriented and communication-oriented, in order to provide consistent and sound dental care. This involves defining the factors influencing oral health in dementia and identifying potential risks for poor oral health in the presence of a diagnosis of dementia.

It is generally known that when dementia is diagnosed, there is a decline in overall health as the disease progresses. Whether there is a relationship between the decline in cognitive abilities and the deterioration of patients' oral health is poorly understood. The possible causes underlying this process may be multiple [6]. In addition to a deterioration in independence, a decrease in the frequency, and quality to perform hygiene measures may also negatively affect oral health. The most commonly reported behaviors of people with dementia include rejection of care, aggression and agitation [7]. This can have negative effects on, complicate or hinder the performance of daily oral/denture hygiene and dental care and treatment. Consequently, a change in oral health and other determinants may result. The frequent intake of snacks between meals or, in the case of additionally diagnosed dysphagia, the consumption of food supplements, which are usually very cariogenic [8], should also be examined as possible causes.

The deterioration of motor skills and the increase in the need for care in people with dementia are associated with an increased risk of oral health problems [9, 10]. The oral health of people with dementia is characterized by the presence of more plaque [11] and the resulting oral health problems of the soft tissues [12] compared to people without dementia. Thus, caries has a high prevalence in people with dementia [13–15]. Poor oral health, resulting reduced chewing function and the presence of oral pain can lead to a decrease in quality of life [16–18].

Furthermore, oral health is dependent on the regular utilization of dental services [19–21]. The utilization of dental services is dependent on several factors [22, 23]. In turn, utilization depends on the possibilities in access to dental services [24], which is more difficult for people with dementia and in need of care, and on cognitive status [25]. Access to dental services enables early diagnosis, prevention and clinical treatment of the (oral) diseases such as caries or periodontitis. It is therefore likely that the utilization of dental services by people with dementia is reduced and might have a negative impact on oral health.

The aim of this study is to show the influence of different degrees of cognitive impairment and dementia on oral health and the utilization of dental services.

## Methods

### Study design

The data of this analyzation is part of the OrBiD (Oral Health, Bite Force and Dementia) pilot study (ClinicalTrials.gov NCT03775772). This OrBiD study was divided into two parts. Part A focused on the investigation of oral health in people with and without dementia, while Part B investigated the chewing function as a function of the degree of dementia. Analyzation of both parts of the OrBiD study took place each at baseline (cross-sectional analysis with inter-cohort analysis) and at a longitudinal evaluation point after the implementation of interventions for each study part. This analysis focuses on the evaluation of cross-sectional data for oral health in people with and without dementia.

Subjects were required to fulfill the following inclusion criteria: Subjects needed to be 60 years of age or older. They were included regardless of their cognitive abilities. Subjects with any acute dental/oral condition (e.g., pain, abscesses) or problems with their temporomandibular joints or surrounding chewing muscles were excluded. It was a requirement that they should have had no or at most one dental hygiene session in the last 12 months before the start of the study participation. Subjects who are edentulous both in the upper and lower jaw were excluded from participation. Subjects with intolerance/allergy to toothpaste with high fluoride content (Duraphat® toothpaste 5000 ppm, Colgate™) and/or phenylalanine were excluded.

Recruitment was randomly done within the patient population of a clinic specialized in gerodontology, or in cooperating facilities (long-term care facilities, geronto-psychiatric facilities). All subjects participated voluntary in the study. Informed consent was obtained from the subjects themselves or their legal guardians.

Subjects were stratified into five dementia evaluation groups (1—no dementia (noDem, MMSE 28–30), 2—mild cognitive impairment (mCI, MMSE 25–27), 3—mild dementia (mDem, MMSE 18–24), 4—moderate dementia (modDem, MMSE 10–17), and 5—severe dementia (sDem, MMSE < 10)) on the basis of the Mini Mental State Examination (MMSE) [5].

### Measurements

Socio-demographic items (age (in years), sex (categories: male. female), living situation (categories: community-dwelling, long-term care facility)) were recorded. All clinical examinations were performed by a single investigator.

I. Effects of the physiological changes of old age

The effects of the physiological changes of age are studied with the following instruments:

- The Barthel-Index of Activities of daily living [26] which is used to systematically record independence or the need for care (maximum score 100 (no need of care)) was recorded.
- The nutritional status was recorded using the Mini Nutritional Assessment (MNA) [27] (maximum score 30 points). An MNA score of 17–23.5 points indicates being at risk for malnutrition. Less than 17 points indicate a poor nutritional status.
- The Oral functional capacity (OFC) was used to assess subjects with regard to their Resilience capacity level (RCL) within the three parameters therapeutic capability, oral hygiene ability and self-responsibility. The levels of the parameter’s therapeutic capability and oral hygiene ability range from 1—normal to 2—slightly reduced, 3—greatly reduced and 4—none. Self-responsibility was recorded with the capacity levels normal, reduced and none. The highest value of one of the three parameters determines the patient's RCL [28].
- Uptake of dental services—The number of visits to a dentist or oral hygienist per year, two years prior the participation in the study, was recorded for each subject.
- The presence of dentures (categories: no denture available/used, removable denture, fixed denture, combination of removable and fixed) and the type of denture (categories: complete denture, model cast prosthesis, temporary denture/molded clamp, temporary denture, denture/precision attachment, telescopic denture, hybrid denture) were recorded separately for the upper and lower jaw.

II. Change in oral health in relation to cognitive impairment

The change in oral health due to the extent of cognitive impairment was measured with the following parameters:

- caries experience:
  The DMF/T index (D—decayed, M—missing, F—filled, T—teeth) is a measure of caries experience. It was used in this study with the knowledge that it is impossible to determine whether a tooth was lost due to caries or other reasons (e.g. trauma, periodontal disease) and related to 32 teeth [29]. The degree of restoration (in percent, %) is calculated from components of the DMF/T Index as follows: (F/D+F) x 100.b)
- periodontal disease:
  The Periodontal Screening Index (PSI) [30] was collected as the worst value per sextant (code 0—healthy, code 1—gingivitis without calculus/plaque and without defective restoration margins, code 2—gingivitis with calculus and/or plaque and/or defective restoration margins, code 3—moderate periodontitis, code 4—severe periodontitis) using a WHO periodontal probe.
  The Bleeding-on-Probing-Index (BOP) (categorical variable: yes/no) [31] indicates the presence of bleeding caused by gentle manipulation of tissue at the depth of the gingival sulcus or the gingiva-to-tooth interface. If at least one site in the maxilla or mandible where probing depths have been measured with a probe was positive (rating "yes"), BOP was rated as positive presence/yes overall.
- oral and denture hygiene:
  The Oral hygiene index (OHI, Greene and Vermillion) was used to quantify oral hygiene. It is calculated as the sum of the two components, debris index (DI) and calculus index (CI). The higher the OHI (possible range 0-12), the worse the oral hygiene. DI and CI can each take a range of 0-6 [32].
  Denture hygiene was assessed visually, without staining by the dentist (categories: no dental plaque, plaque on the outer or inner surface of the denture, calculus on the outer or inner surface of the denture).

### Statistical considerations

Descriptive analysis methods were used where indicated. Quantitative variables are given as median, range, mean and standard deviation (SD). Qualitative variables were evaluated as absolute and relative frequencies. Pearson`s Chi-Square test was used to determine statistically significant differences between the expected and observed frequencies between the categories of the variables.

The Kruskal–Wallis test was used to determine differences in the central tendencies of several independent samples. The Jonckheere-Terpstra test was used in the same way as the Kruskal–Wallis test but considering the priori ordering of the population in this study by means of the MMSE value grouping. The *p*-values of all statistical tests used were interpreted strictly descriptively. The significance level was set at α = 0.05. Statistical tests were not performed for variables with low expected values. The statistical analysis was done with IBM`s SPSS Version 23.0[33].

### Ethical considerations

The study was approved by the competent Cantonal Ethics Committee (CEC) of Zurich (KEK-ZH 2017-00363). All subjects or their legal representatives gave written informed consent.

## Results

### Study population

A total of 137 subjects, who were assigned to five evaluation groups (group 1—noDem, group 2—mCI, group 3—mDem, group 4—modDem, group 5—sDem) based on their Mini Mental State examination values, were included in the analysis. With the increase in cognitive impairment, the age of the subjects (*p* = 0.001, Jonckheere Terpstra) and the proportion of subjects cared for in a long-term care facility (*p* = 0.001, Jonckheere Terpstra) increased significantly. The proportion of women in the evaluation groups with cognitive impairments or dementia (evaluation groups mCI to sDem) was 60–75% (Table 1).

**Table 1:** Subjects characteristics for socio-demographic, geriatric assessment items and the oral functional capacity by evaluation group

	noDem MMSE 28–30 (n = 26)	mCI MMSE 25–27 (n = 29)	mDem MMSE 18–24 (n = 27)	modDem MMSE 10–17 (n = 30)	sDem MMSE < 10 (n = 25)
*Socio-demographic items*					
Age (years)					
Median (Range)	75 (62–92)	80 (61–95)	86 (65–95)	88 (61–99)	87 (67–97)
Sex (n/%)					
Female	13/50	18/62.1	20/74.1	21/70	18/72
Living situation (n/%)					
Community-dwelling	23/88.5	19/65.5	6/22.2	0/0	0/0
Long-term care facility	3/11.5	10/34.5	21/77.8	30/100	25/100
*Geriatric items*					
Barthel index Median (Range)	(n = 26) 100	(n = 29) 100 (30–100)	(n = 27) 90 (25–100)	(n = 22) 30 (10–100)	(n = 19) 20 (0–65)
MNA Normal (MNA 24–30) At risk (MNA 17–23.5) Malnourished (MNA < 17)	(n = 26) 21/80.8 3/11.5 2/7.7	(n = 29) 18/62.1 11/37.9 0/0	(n = 26) 9/34.6 15/57.7 2/7.7	(n = 22) 3/13.6 15/68.2 4/18.2	(n = 21) 0/0 12/57.1 9/42.9
*OFC*					
TC (n/%)					
Normal Slightly reduced Greatly reduced None	20/76.9 6/23.1 0/0 0/0	14/48.3 15/51.7 0/0 0/0	1/3.7 9/33.3 16/59.3 1/3.7	0/0 2/6.7 21/70.0 7/23.3	0/0 1/4.0 8/32.0 16/64.0
OHA (n/%)					
Normal Slightly reduced Greatly reduced None	16/61.5 9/34.6 1/3.8 0/0	6/20.7 19/65.5 4/13.8 0/0	1/3.7 7/25.9 16/59.3 3/11.1	0/0 2/6.7 20/66.7 8/26.7	0/0 0/0 8/32.0 17/68.0
SR (n/%)					
Normal Reduced None	24/92.3 2/7.7 0/0	21/72.4 8/27.6 0/0	2/7.4 11/40.7 14/51.9	0/0 11/36.7 19/63.3	0/0 0/0 25/100
RCL (n/%)					
1 2 3 4	14/53.8 11/42.3 1/3.8 0/0	5/17.2 18/62.1 6/20.7 0/0	1/3.7 1/3.7 11/40.7 14/51.9	0/0 0/0 11/36.7 19/63.3	0/0 0/0 0/0 25/100.0
*Uptake of dental services* Number of visits in the last two years prior to study participation (number of patients observed)					
Dentist Median (Range)	(n = 26) 1 (0–4)	(n = 27) 2 (0–3)	(n = 26) 2 (0–3)	(n = 27) 1 (0–3)	(n = 22) 1 (0–4)
Dental hygienist Median (Range)	(n = 26) 3 (0–7)	(n = 27) 3 (0–5)	(n = 25) 2 (0–5)	(n = 26) 1 (0–3)	(n = 22) 0 (0–6)

*MMSE* Mini mental state examination; *MNA* Mini nutritional assessment; *OFC* oral functional capacity, *TC* therapeutic capability, *OHA* oral hygiene ability, *SR* self-responsibility, *RCL* resilience capacity level, *noDem* no dementia, *mCI* mild cognitive impairment, *mDem* mild dementia, *modDem* moderate dementia, *sDem* severe dementia

### Effects of the physiological changes of old age

There were differences between the evaluation groups in the Barthel index, which worsened with the increase in cognitive deficits (Table 1).

**Fig. 1:**
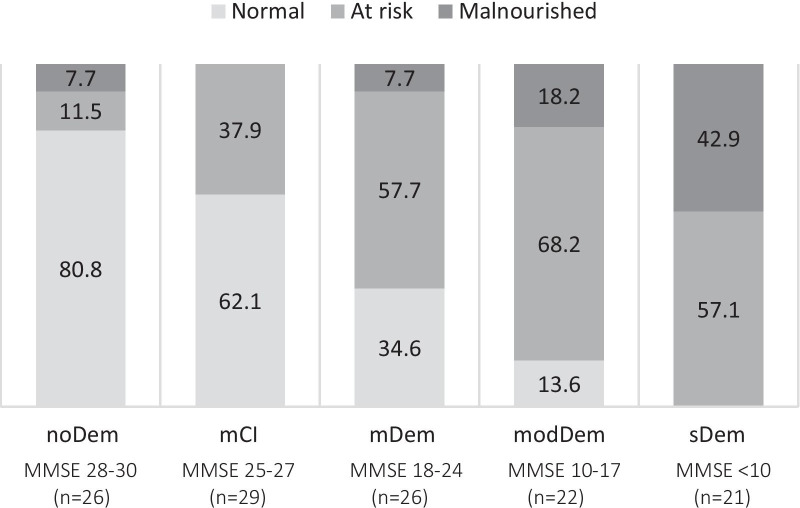
Mini nutritional assessment (MNA) in the categories normal (MNA score 24–30), at risk (MNA score 17–23.5), and malnourished (MNA score < 17) for each evaluation group in percent (%). *noDem* no dementia, *mCI* mild cognitive impairment, *mDem* mild dementia, *modDem* moderate dementia, *sDem* severe dementia)

The subjects differed in their risk of suffering from malnutrition. In subjects of the evaluation group noDem, the majority of subjects were well nourished (MNA normal 80.8%). This proportion decreased with the increase in cognitive impairment, while the proportion of subjects at risk or with malnutrition, measured by MNA values, increased (Table 1, Fig. 1).

OFC parameters (therapeutic ability, oral hygiene ability and self-responsibility) as well as the overall RCL differed between the evaluation groups. With the increase in dementia, a deterioration of the OFC parameters and the RCL was evident (Table 1).

The evaluation groups differed significantly in their utilization of dental services, both in the utilization of the dentist (*p* = 0.004, Kruskal–Wallis) or the dental hygienist (*p* = 0.001, Kruskal–Wallis). The number of visits to the dentist was almost the same for subjects with noDem, mCI and mDem, but decreased with the increase in dementia. This decrease in demand was particularly noticeable in the utilization of the dental hygienist. A continuous decrease in the utilization of the dental hygienist with the increase in dementia up to evaluation group modDem was observed (Table 1).

The majority of all subjects had removable dentures in the upper jaw (exception evaluation group mDem 29.6% removable dentures, 55.6% fixed dentures) (noDem 73.1%, mCI 48.3%, modDem 43.3%, sDem 40%). Only subjects with noDem wore removable dentures in the lower jaw (n = 15, 57.7%). Subjects with mCI and sDem had predominantly no dentures (mCI n = 10, 34.5%; sDem n = 9, 36%), subjects with mDem and modDem had predominantly fixed dentures (mDem n = 13, 48.1%; modDem n = 14, 46.7%) in the lower jaw.

The existing removable dentures in the maxilla were mostly clasp retained removable dentures (noDem 55%, mCI 64.2%, mDem 50%, modDem 46.2%; exceptions: presence of total dentures in the maxilla modDem 46.2%, sDem 70%). In the lower jaw the removable denture was mostly clasp retained (noDem 81.4%, mCI 60%, modDem 57.2%; exceptions: presence of total dentures in the upper jaw modDem 46.2%, sDem 70%).

### Change in oral health in relation to cognitive impairment

- DMF/T and degree of restoration

As a function of dementia, there was no difference between the evaluation groups in the DMF/T index and its individual components in terms of the number of teeth missing (MT, *p* = 0.152, Kruskal–Wallis), filled (FT, *p* = 0.075, Kruskal–Wallis) and filled or decayed (DF, *p* = 0.079, Kruskal–Wallis). There was a significant difference between the evaluation groups with regard to the number of teeth that are decayed (DT) (*p* = 0.001, Kruskal–Wallis) (Table 2).

**Table 2:** DMF/T index (related to 32 teeth) and its components separated by evaluation group. (*p* < 0.05)

		noDem MMSE 28–30 (n = 26)	mCI MMSE 25–27 (n = 29)	mDem MMSE 18–24 (n = 27)	modDem MMSE 10–17 (n = 30)	sDem MMSE < 10 (n = 25)	*p*
Tooth status	Index	Median (Range) Mean ± SD	Median (Range) Mean ± SD	Median (Range) Mean ± SD	Median (Range) Mean ± SD	Median (Range) Mean ± SD	
Caries experience	DMFT	28.5 (18–32) 27.8 ± 3.7	27 (17–32) 26.8 ± 3.9	27 (20–32) 26.9 ± 3.2	29.5 (18–32) 27.4 ± 4.7	26 (14–32) 25.9 ± 6.1	0.696

		Median (Range)	Median (Range)	Median (Range)	Median (Range)	Median (Range)	
Decayed teeth	DT	0 (0–1)	0 (0–6)	0 (0–8)	1 (0–7)	0 (0–20)	0.001
Missing teeth	MT	19 (4–29)	15 (4–30)	10 (3–28)	13 (4–31)	13 (0–30)	0.152
Filled teeth	FT	8.5 (3–21)	10 (0–22)	14 (2–24)	11 (0–24)	8 (1–24)	0.075
Decayed-filled teeth	DF	9 (3–21)	13 (1–22)	14 (2–24)	12.5 (1–27)	8 (2–31)	0.079

noDem *no dementia,* mCI *mild cognitive impairment,* mDem *mild dementia,* modDem *moderate dementia,* sDem *severe dementia*

The evaluation groups differed significantly in the degree of restoration (*p* = 0.001, Kruskal–Wallis), which decreased with the increase in dementia (noDem 98.8%, mCI 91.6%, mDem 94.2%, modDem 85.3%, sDem 78.8%) (Table 2).

- Peridontal Screening Index (PSI) and Bleeding on Probing (BOP)

None of the subjects in all evaluation groups showed the codes 0 (healthy) and 1 (reversible gingivitis) in PSI. The majority of all subjects in each evaluation group had periodontal disease (code 3 and 4 moderate/severe periodontitis). The proportion of subjects with gingivitis/calculus present (code 2) in the groups varied between 0 and 19.2% (Table 3).

**Table 3:** Periodontal screening Index (PSI) and associated periodontal status as number of subjects and percentage of subjects for every PSI Code (0–4) separated by evaluation group

		noDem MMSE 28–30 (n = 26)	mCI MMSE 25–27 (n = 29)	mDem MMSE 18–24 (n = 27)	modDem MMSE 10–17 (n = 29) *	sDem MMSE < 10 (n = 17) *
Periodontal status	Highest PSI	n/%	n/%	n/%	n/%	n/%
Gingivitis/calculus present	2	5/19.2	3/10.3	0/0	1/3.4	3/17.6
Moderate periodontitis	3	15/57.7	15/51.7	13/48.1	16/55.2	6/35.3
Severe periodontitis	4	6/23.1	11/37.9	14/51.9	12/41.4	8/47.1

*noDem* no dementia, *mCI* mild cognitive impairment, *mDem* mild dementia, *modDem* moderate dementia, *sDem* severe dementia

The PSI codes 0—healthy and 1—reversible gingivitis are not existent in all subjects (all subjects PSI 0: n = 0, PSI 1: n = 0). * examination not possible in all subjects

Overall, the majority of subjects in all evaluation groups required periodontal therapy (codes 3 and 4) (Table 3).

The evaluation groups differed significantly (*p* = 0.033, Chi2 test) with regard to the parameter BOP. With the increase in dementia, the proportion of subjects with bleeding on probing at at least one PSI measurement site increased (BOP positive: noDem 69.2%, mCI 86.2%, mDem 88.9%, modDem 92.9%, sDem 100%).

- Oral and denture hygiene

The OHI (*p* = 0.001, Jocksteere Terpstra) and its individual parameters debris index (DI, *p* = 0.001, Jocksteere Terpstra) and calculus index (DI, (*p* = 0.001, Jocksteere Terpstra) increased with the increase in dementia and differed highly significantly between the evaluation groups (Table 4).

**Table 4:** Oral hygiene index (OHI) and its components debris index (DI) und calculus index (CI) separated by evaluation group (*p* < 0.05)

Index	noDem MMSE 28–30 (n = 25)	mCI MMSE 25–27 (n = 29)	mDem MMSE 18–24 (n = 27)	modDem MMSE 10–17 (n = 29)	sDem MMSE < 10 (n = 20)	*p*
DI Median (Range)	2 (1–5)	2.7 (0.4–4.5)	3.2 (0.5–5.5)	4 (2–6)	4 (2.3–6)	0.001
CI Median (Range)	1.7 (0–3)	2 (0–4.2)	3 (0.25–4.5)	3 (1.3–6)	3.3 (2–6)	0.001
OHI Median (Range)	3.5 (1–7.7)	4.7 (1–8.7)	6.2 (0.75–9.5)	7.2 (3.3–12)	7 (4.8–12)	0.001

*noDem* no dementia, *mCI* mild cognitive impairment, *mDem* mild dementia, *modDem* moderate dementia, *sDem* severe dementia

**Fig. 2:**
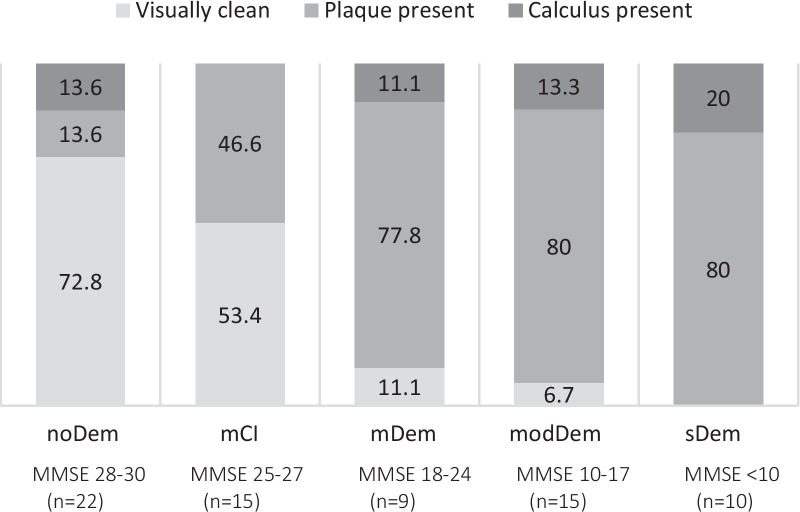
Denture hygiene (categories visually clean, plaque present, calculus present) in percent (%) by evaluation group. *noDem* no dementia, *mCI* mild cognitive impairment, *mDem* mild dementia, *modDem* moderate dementia, *sDem* severe dementia)

The denture hygiene deteriorated significantly with the increase in dementia in the evaluation groups. (*p* = 0.001, Chi2 test) (Fig. 2).

## Discussion

### Study design and restrictions due to the fact that the study participants are suffering from dementia

The study had several limitations mostly because of the inherent difficulties in investigating people with dementia.

The simple, time-efficient and often used Mini Mental State Examination (MMSE) [5] is considered as a suitable instrument to assess cognitive impairment in medical practice. It can be used as a basic diagnostic tool for the preliminary quantification of cognitive deficits and as assessment of their severity [34] but the sensitivity is questionable, especially in mild dementia. The use of the MMSE, instead of more comprehensive testing procedures, may have led to inaccuracies in the grouping of some subjects.

The degree of dementia and the setting during the evaluation (e.g. clinic vs. long-term care facility) may influence the compliance and cooperation of the subjects during the oral health assessment.

It can be assumed that some measurements (e.g. detection of caries and periodontitis) are more prone to misinterpretation than others (e.g. Oral hygiene index (OHI)). Experience shows that measurement procedures that are associated with patience and the acceptance of inconvenience (e.g. probing of pocket depth) are not well tolerated with the increase in cognitive impairment. To the authors best knowledge there are no studies investigating the reliability of the used measurement instruments in people with dementia or different evaluation settings (dental chair vs. examination in a long-term care facility without a dental chair and its light source, most of the subjects sitting upright or lying in bed etc.).

Although the investigator was a dentist experienced in patients with dementia, not all measurements could be completed during the evaluation due to incompliance or other unfavorable circumstances.

As a result, some parameters in the analyzed data set showed expectation values that were too low, which allowed a statistical evaluation only on a purely descriptive basis (e.g. Periodontal Screening Index (PSI), Bleeding on Probing (BOP)).

Most subjects with mild, moderate or severe dementia are long-term care residents, required comprehensive care and had been examined in the long-term care facility. For the recruitment of subjects with advanced dementia, we have had a limited number of long-term care facilities that cooperate with both the inpatient and mobile dental clinic (mobiDent™). Subjects without dementia or with mild cognitive impairment, living at home, were recruited within the clientel of the dental clinic and examined there.

### Utilization of dental services

The utilization of dental services was found to be dependent on the degree of dementia. With the increase in dementia, there was a significant reduction in the demand on the services of the dentist and dental hygienist.

Despite the possibility for the uptake of dental services with the mobile dental clinic for subjects of this study living in long-term care facilities, the utilization of dental services by a dentist and especially by a dental hygienist decreased significantly with increasing dementia. It can therefore be assumed that the utilization of dental services by people with dementia depends more on the cognitive abilities, the need for care, the living situation and the oral functional capacity than on the possible access to dental services. The influence of these and other factors on the use of dental services by people living in long-term care facilities should be observed in further studies to adapt the concepts of mobile dental services offered.

Chen et al. (2013) showed that oral health measurements are poor in people with dementia independent of their residential status. They assumed that oral health had declined before the change in the living situation [35]. Therefore our recruiting process should not have any influence on the results. Warren et al. (1997) conclude that it is unclear when the effects of a cognitive decline, the loss of social support and the functional decline cumulate and combine as risk factors for a deteriorated oral health. Furthermore, this effect may arise only in later stages of dementia and shows an individual variety [13].

### Oral health

In our study there was a significant difference in the number of teeth that are decayed, but not in the number of filled or missing teeth or the DMF/T index itself with increasing cognitive impairment or dementia. A dependency was found between the degree of restoration and dementia, which decreases with increasing dementia. Oral and denture hygiene deteriorated significantly with the increase in dementia. Nevertheless, periodontal therapy was required for all subjects independent of their degree of dementia while bleeding on probing was increasing with increasing dementia.

There was no difference in the DMFT index and the number of missing or filled teeth while a significant difference in the number of decayed teeth was observed with regard to the different dementia groups in this study. In the literature no differences in the DMFT mean values are described also [36, 37]. An explanation for this finding might be that people with dementia received the same dental treatment as people without dementia before the onset and progression of the disease and the accompanied increase in need of care. Nevertheless, Ribeiro et al. report a higher DMFT in subjects with Alzheimer`s disease [38]. Furthermore, caries is more common in people with dementia [13–15] which confirms our findings. A cause for this finding might be the increasing difficulties or inability to treat people with progressed dementia chairside. Often only treatments under general anaesthesia (GA) are possible [39]. If relatives/legal representatives, medical or financial circumstances do not allow a treatment under GA this might result in a higher number of decayed teeth in people with dementia.

The proportion of subjects with moderate and severe periodontitis was high in this study and did not differ with regard to the degree of dementia. Several studies in the literature confirm our results by showing no significant differences in gingival or periodontal diseases between people with and without dementia [36, 40–44]. An explanation might be that the prevalence of progressed periodontal diseases is high in older patients. Periodontal diseases might therefore have been established in people with dementia before the onset of the disease which complicates the evaluation of the influence of dementia on periodontal diseases. It is conceivable that oral health, in general, might have already deteriorated in the frailty phase of life, regardless of cognitive impairment. On contrary, De Souza et al. (2014) point out that people with dementia suffered more from periodontal disease than people without dementia [45].

With increasing dementia, Bleeding in Probing (BOP) increased significantly in this study as described before by Maldonado et al. (2017) [46].

The Oral Hygiene Index (OHI) and its components (Debris Index (DI), Calculus Index (CI)) by Greene and Vermillion increased with the increase in dementia and differed highly significantly between the dementia groups in this study. In the literature there are studies using the OHI index by Greene and Vermillion which report a Debris Index (DI) of 2.1, a Calculus Index (CI) of 2.0 and an OHI of 4.5. in subjects with dementia [38, 47, 48]. which results in a better oral hygiene of the subjects than in our study (mDem: DI Median 3.2 (Range 0.5–5.5), CI Median 3 (Range 0.25–4.5), OHI Median 6.2 (Range 0.75–9.5); modem: DI Median 4 (2–6), CI Median 3 (1.3–6), OHI Median 7.2 (Range 3.3–12); sDem: DI Median 4 (Range 2.3–6), CI Median 3.3 (Range 2–6), OHI Median 7 (Range 4.8–12)). Furthermore, Warren et al. (1997) observed no significant differences between dementia subtypes and healthy controls for the DI but investigated significant differences in the DI between people with moderate to severe dementia compared to people without dementia [13].

The denture hygiene, which was assessed visually without staining by the dentist, deteriorated significantly with the increase in dementia in this study. Zenthöfer et al. (2014) observed no differences in the denture hygiene between people with and without dementia measured with the Denture Hygiene Index (DHI) [11]. Due to the nature of the measurements in the study of Zenthöfer et al. (2014) and ours no comparison of the results are possible. Since in our study, the utilization of dental services was lower for people with dementia, this might explain the worse denture hygiene observed. Also, a bias cannot completely be ruled out since the investigator in this study was not blinded. Furthermore, the visual judgement has limitations in differentiating between different hygiene status`.

The degree of restoration decreased significantly with the increase in dementia which might be a consequence of the reduced utilization of dental services. Also, one must consider that dental treatments in patients with dementia and an associated reduced Oral functional capacity (OFC) might have be limited due to reduced compliance or the avoidance of treatments under general anaesthesia, for example. In the literature no findings about the degree of restoration in people with dementia can be found to the authors best knowledge.

Since periodontal disease were common in all subjects independently of their cognitive status, but BOP, as an acute sign of inflammation, and the number of decayed teeth was higher in people with dementia, a higher impact of the daily oral hygiene might be considered as an explanation. Therefore, the authors suggest to substantially improve daily oral hygiene of people with dementia to reduce plaque and the resulting oral diseases (periodontitis, caries). A prerequisite for this is a structured training of nursing staff and relatives who are involved with daily oral and denture hygiene. This also includes intensive practical exercises which should be carried out by dental professionals. This requires structured concepts, which might also need to be co-financed by the health insurance companies as part of a prevention promotion program. In addition to improving the training and further education of nursing staff, individual hygiene concepts as well as education in nutritional aspects (less cariogenic food, reduced frequency of (finger)food etc.) that a dentist or dental hygienist could develop and implement with nursing staff, carers and relatives should also be financially supported by third parties (e.g. health insurance companies) since the deterioration in oral health seems to be mainly a consequence of the cognitive decline.

Interdisciplinary research cooperation’s should be strengthened in order to facilitate recruitment processes and improve research in people with dementia.

## Conclusions

Overall, this study was able to show an influence of cognitive impairment and dementia on both, oral health and on the utilization of dental services. Further longitudinal studies are needed to draw conclusions about the influence of the utilization behavior of people with dementia on specific oral health parameters.

To improve the theoretical knowledge of nursing staff, practical oral hygiene concepts for institutions and individual patients also considering nutritional aspects (reduced frequency and less cariogenic foods, etc.) should be established. Dentists/dental hygienists could develop these concepts for the facilities together with the managers, all professional groups of the nursing home and relatives. Care should be supported in a structured way, as the deterioration of oral health seems to be mainly a consequence of cognitive decline. Interdisciplinary research collaboration should be strengthened to facilitate recruitment processes and improve research on and for people with dementia.

## Data Availability

The datasets used and/or analysed during the current study are available from the corresponding author on reasonable request.

## Abbreviations

BOP: Bleeding on probing
CI: Calculus Index
DHI: Denture Hygiene Index
DI: Debris Index
DMFT index: Decayed-, Missing-, Filled- Teeth index
MMSE: Mini Mental State Examination
MNA: Mini Nutritional Assessment
OFC: Oral functional capacity
OHI: Oral Hygiene Index
OrBiD study: Oral Health, Bite force and Dementia Study
PSI: Peridontal Screening Index
RCL: Resilience capacity level
